# Supplementary material for: B4GALT1 Is a New Candidate to Maintain the Stemness of Lung Cancer Stem Cells
Source: J Clin Med. 2019 Nov 9;8(11):1928. doi: 10.3390/jcm8111928 (PMC6912435; doi:10.3390/jcm8111928)
Supplement: Supplementary file 1 [file jcm-08-01928-s001.zip › supplementary/supplementary figures and Table S1.docx]

**
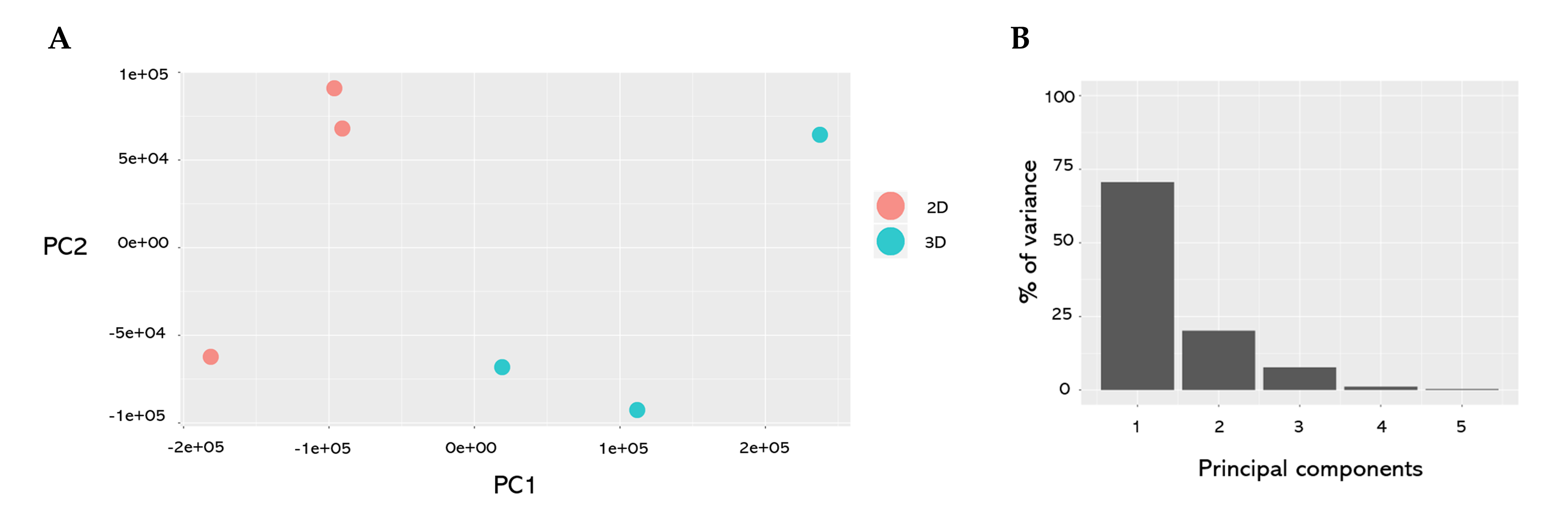
**

**Figure S1. Quality Control of RNA-seq.** (**A**) PCA analyses of RNA-seq profiled 2D and 3D cultures of NCI-H460 cell line (red = 2D; blue = 3D). (**B**) The fraction of variance explained by the principal components.


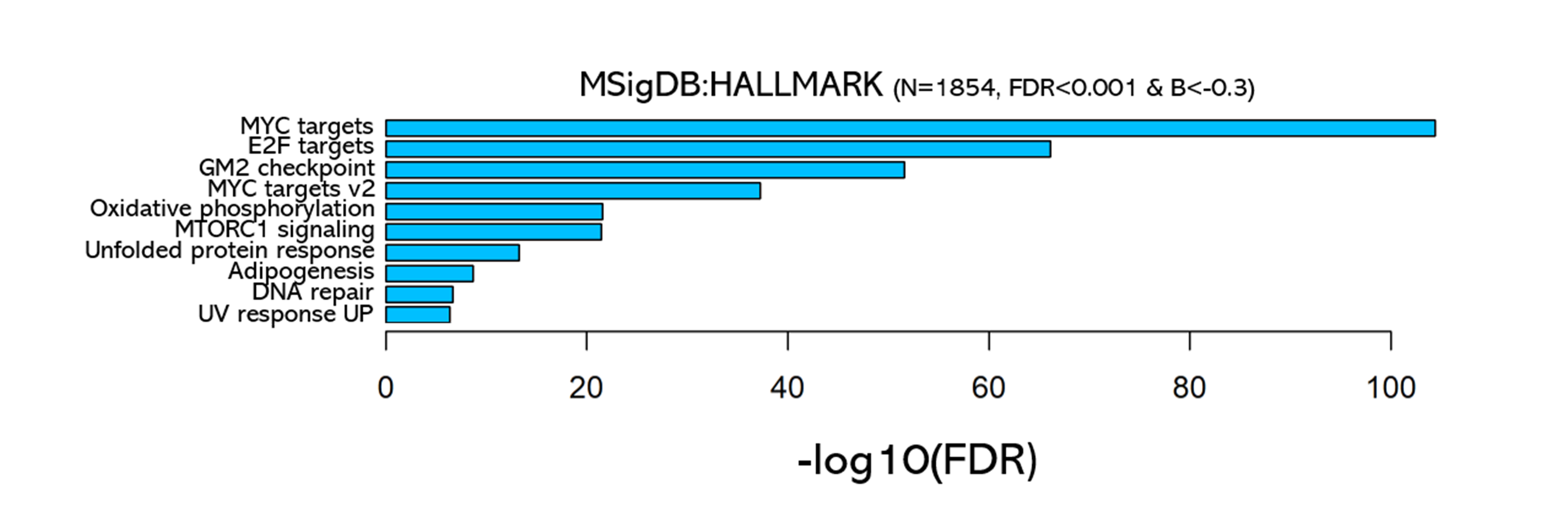


**Figure S2. MSigDB: HALLMARK enrichment of the downregulated genes in 3D vs. 2D cultures.**

**
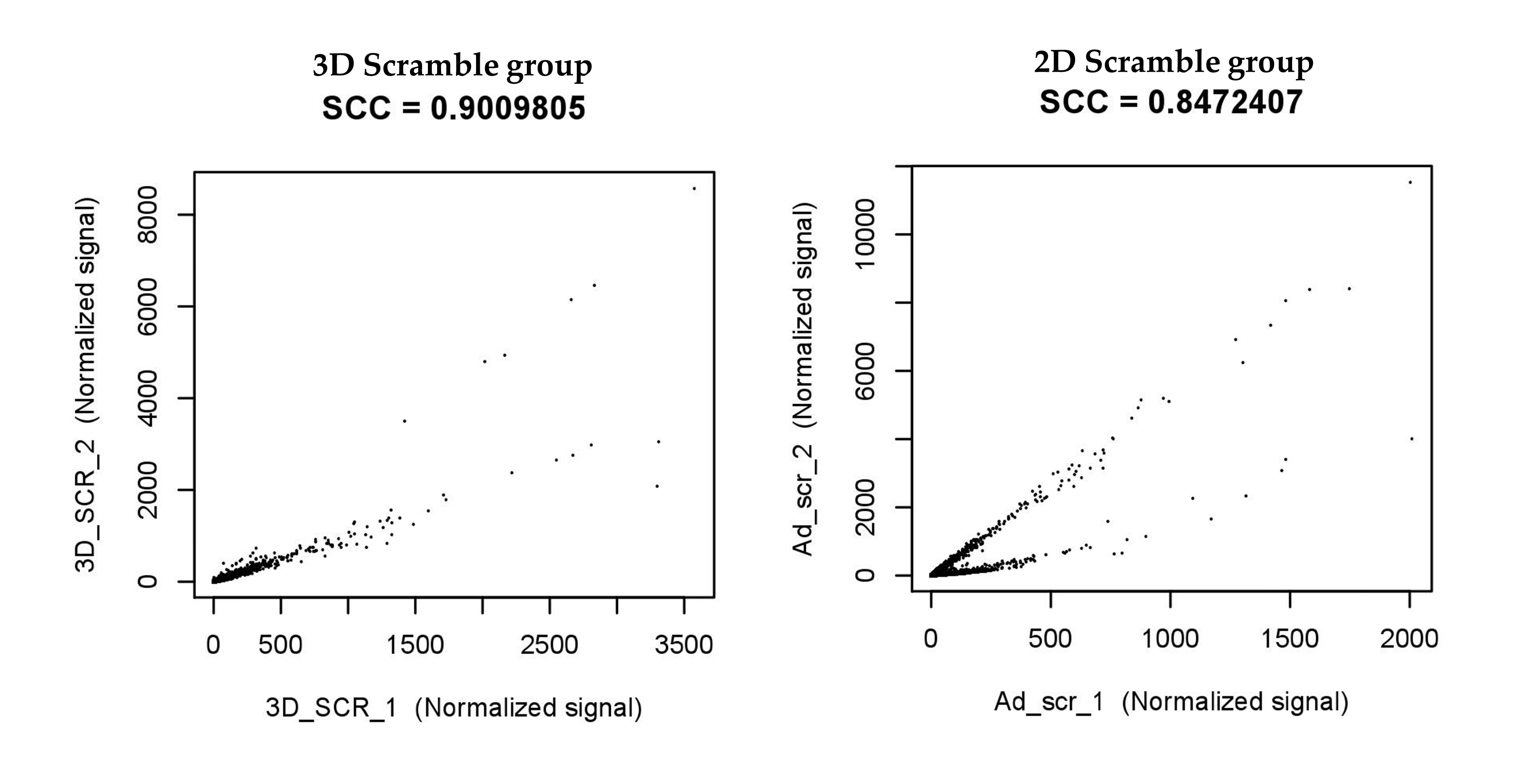
**

**Figure S3. Quality Control of ATAC-seq.** Spearman rank correlation of ATAC-seq duplicates. SCC = Spearman's rank correlation coefficient.


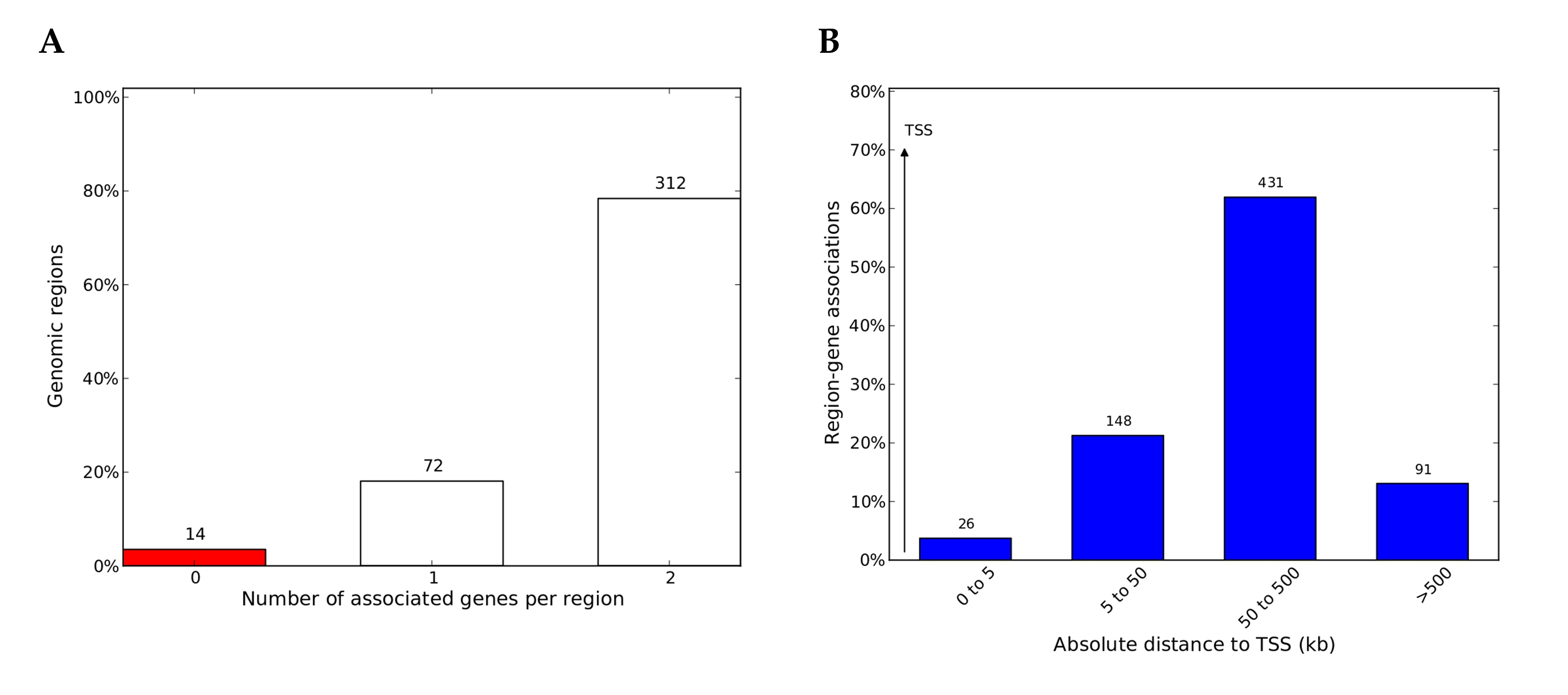


**Figure S4.** **Differential significant ATAC-seq sites in relationship with the relative genomic neighbourhood.** (**A**) The number of genes putatively associated by bps distance (within a window of 500kb) to each differential regulatory region. (**B**) The number of differential regulatory sites in the function of the absolute distance to the closest gene TSS.


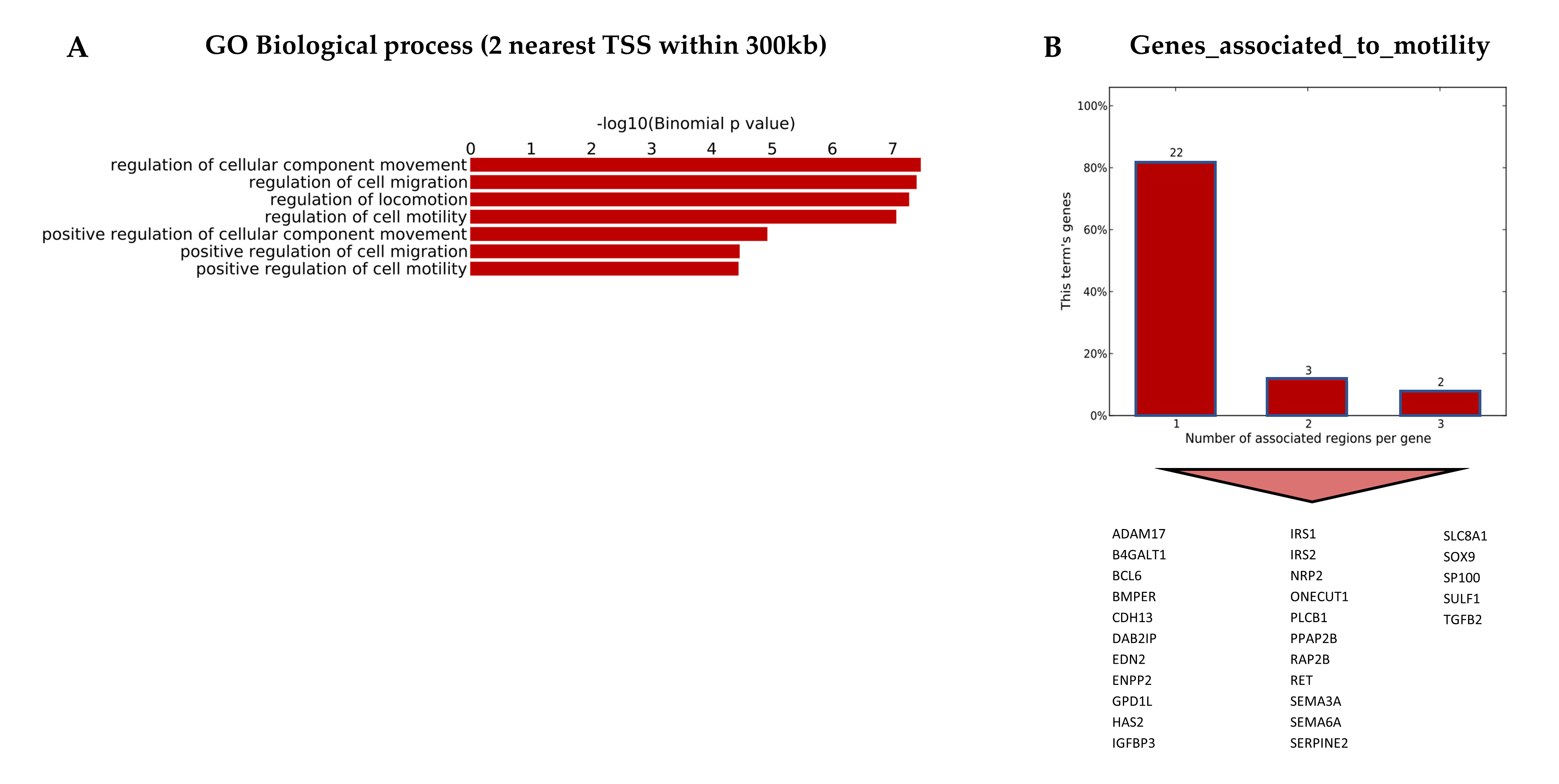


**Figure S5. Enrichment analysis of top 100 up-regulated sites in 3D vs 2D ATAC-seq.** (**A**) Gene Ontology Biological processes considering the two nearest TSS within a window of 300kb from the selected regulatory regions. (**B**) Barplot depicting the number of genes associated with “motility” to each regulatory region. Below the gene symbol of genes associated with motility.


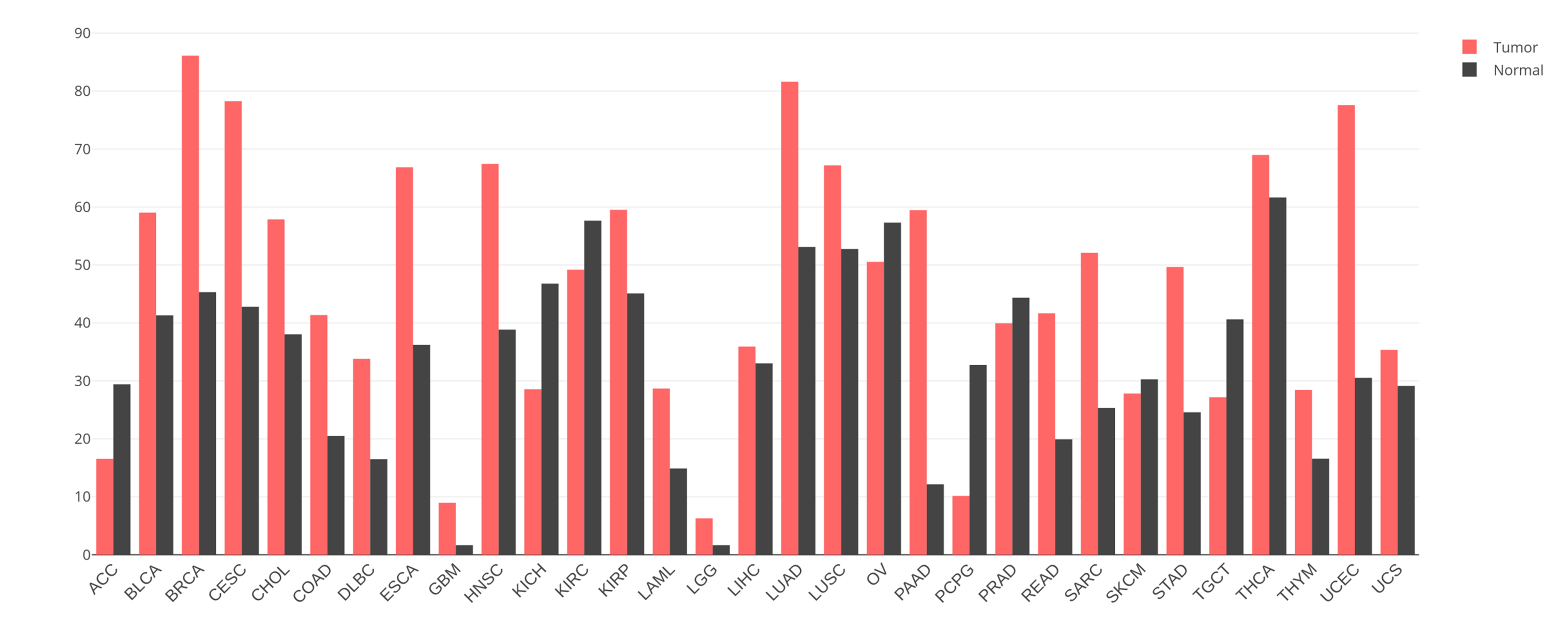


**Figure S6. PanCancer analysis of B4GALT1 expression.** The barchart represents the B4GALT1 expression in 31 cancer types and the relative normal (red = cancer; grey = normal). Abbreviations = ACC Adrenocortical carcinoma; BLCA Bladder Urothelial Carcinoma; BRCA Breast invasive carcinoma; CESC Cervical squamous cell carcinoma and endocervical adenocarcinoma; CHOL Cholangio carcinoma; COAD Colon adenocarcinoma; DLBC Lymphoid Neoplasm Diffuse Large B-cell Lymphoma; ESCA Esophageal carcinoma; GBM Glioblastoma multiforme; HNSC Head and Neck squamous cell carcinoma; KICH Kidney Chromophobe; KIRC Kidney renal clear cell carcinoma; KIRP Kidney renal papillary cell carcinoma; LAML Acute Myeloid Leukemia; LGG Brain Lower Grade Glioma; LIHC Liver hepatocellular carcinoma; LUAD Lung adenocarcinoma; LUSC Lung squamous cell carcinoma; MESO Mesothelioma; OV Ovarian serous cystadenocarcinoma; PAAD Pancreatic adenocarcinoma; PCPG Pheochromocytoma and Paraganglioma; PRAD Prostate adenocarcinoma; READ Rectum adenocarcinoma; SARC Sarcoma; SKCM Skin Cutaneous Melanoma; STAD Stomach adenocarcinoma; TGCT Testicular Germ Cell Tumors; THCA Thyroid carcinoma; THYM Thymoma; UCEC Uterine Corpus Endometrial Carcinoma; UCS Uterine Carcinosarcoma; UVM Uveal Melanoma.


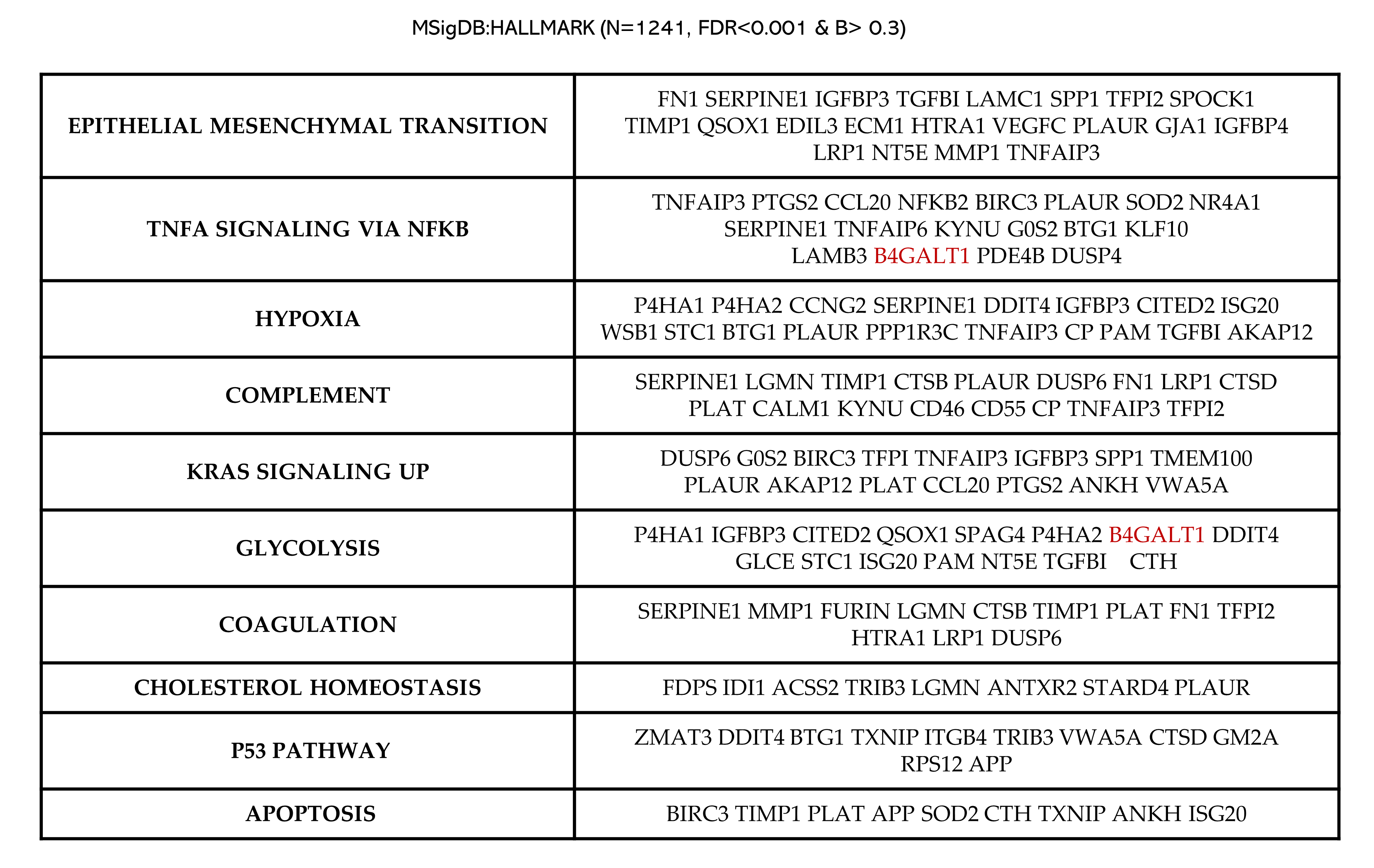


**Table S1. Upregulated genes in 3D vs. 2D associated with most significant MSigDB: HALLMARKs.**
