# Supplementary material for: B4GALT1 Is a New Candidate to Maintain the Stemness of Lung Cancer Stem Cells
Source: J Clin Med. 2019 Nov 9;8(11):1928. doi: 10.3390/jcm8111928 (PMC6912435; doi:10.3390/jcm8111928)
Supplement: Supplementary file 1 [file jcm-08-01928-s001.zip › supplementary/supplementary figures and Table S1.pdf]

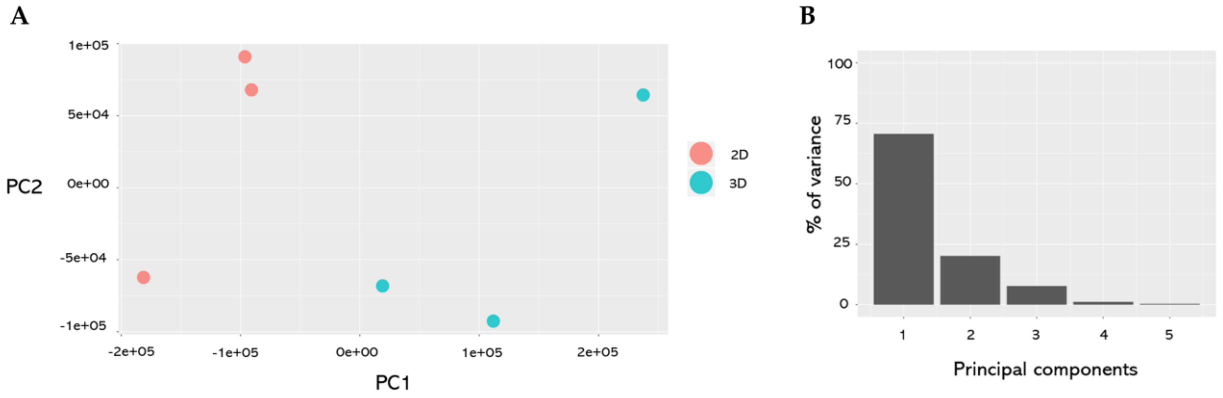

Figure S1. Quality Control of RNA-seq. (**A**) PCA analyses of RNA-seq profiled 2D and 3D cultures of NCI-H460 cell line (red = 2D; blue = 3D). (**B**) The fraction of variance explained by the principal components.

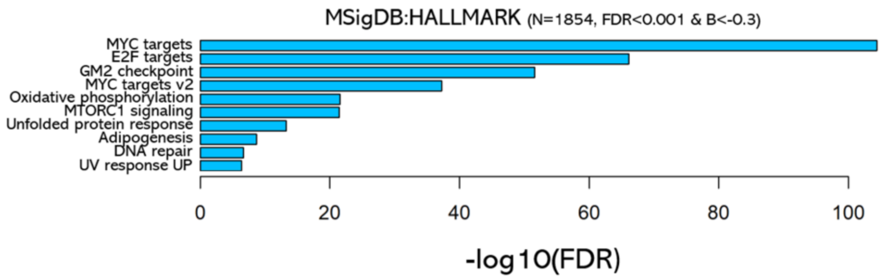

Figure S2. MSigDB: HALLMARK enrichment of the downregulated genes in 3D vs. 2D cultures.

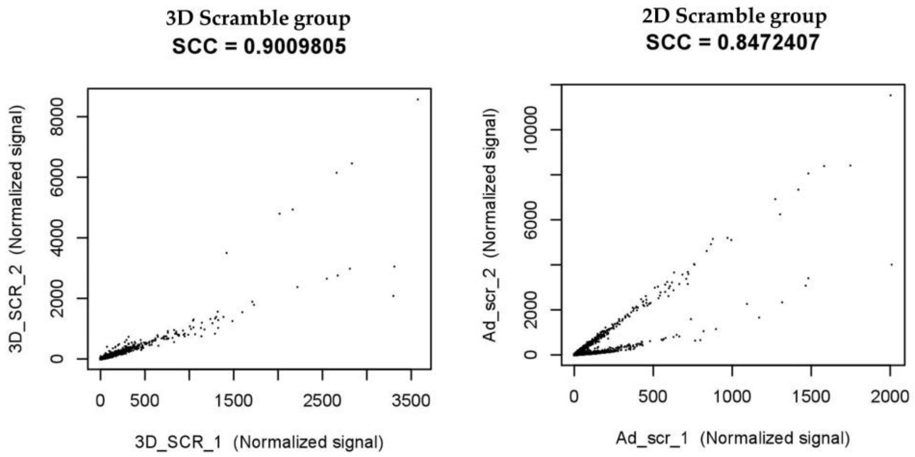

Figure S3. Quality Control of ATAC-seq. Spearman rank correlation of ATAC-seq duplicates. SCC = Spearman's rank correlation coefficient.

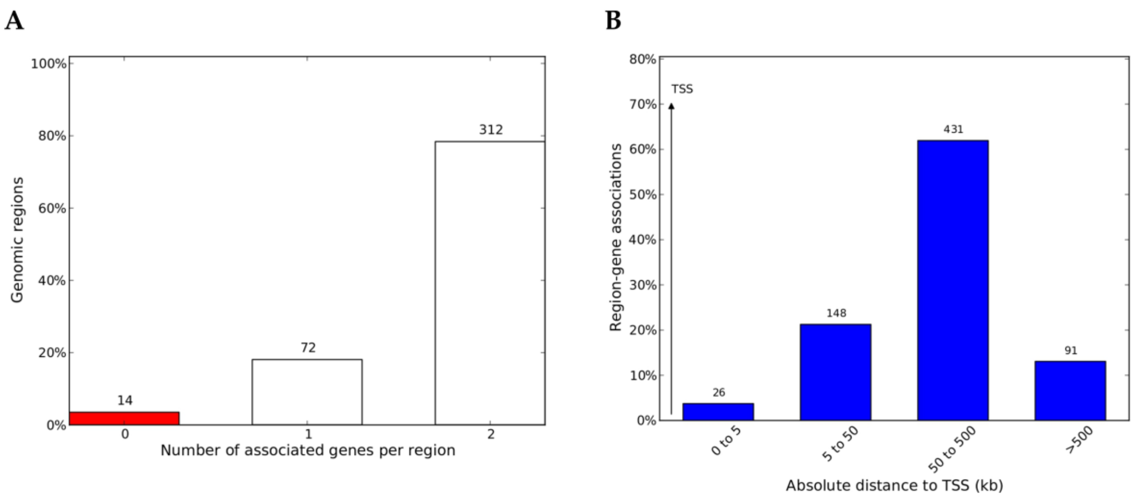

Figure S4. Differential significant ATAC-seq sites in relationship with the relative genomic neighbourhood. (**A**) The number of genes putatively associated by bps distance (within a window of 500kb) to each differential regulatory region. (**B**) The number of differential regulatory sites in the function of the absolute distance to the closest gene TSS.

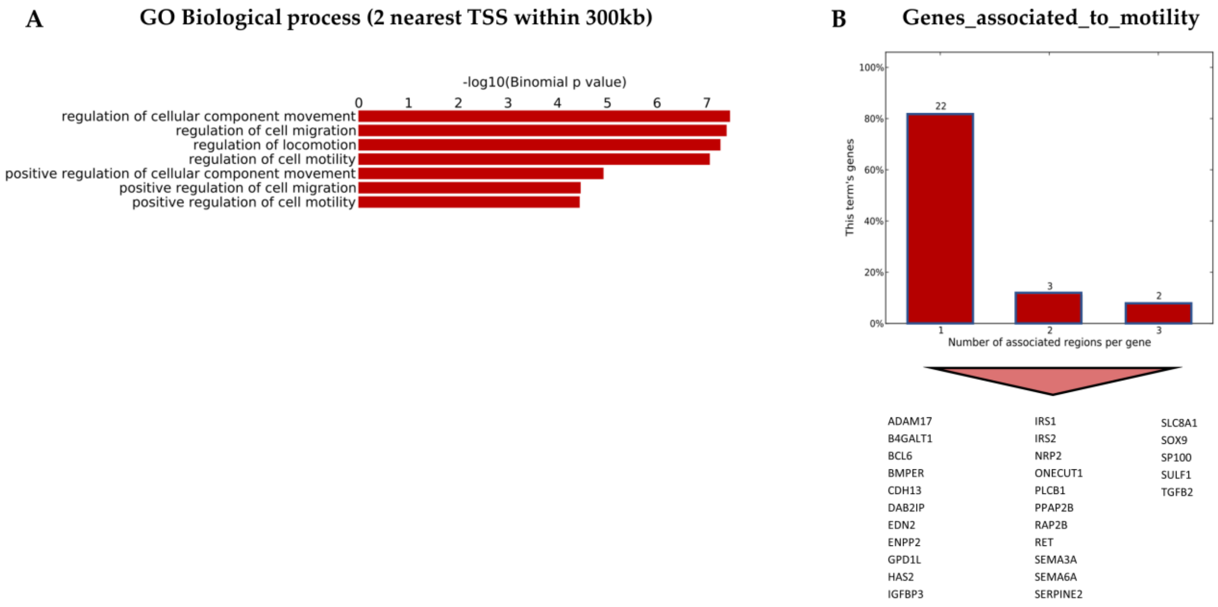

Figure S5. Enrichment analysis of top 100 up-regulated sites in 3D vs 2D ATAC-seq. (**A**) Gene Ontology Biological processes considering the two nearest TSS within a window of 300kb from the selected regulatory regions. (**B**) Barplot depicting the number of genes associated with "motility" to each regulatory region. Below the gene symbol of genes associated with motility.

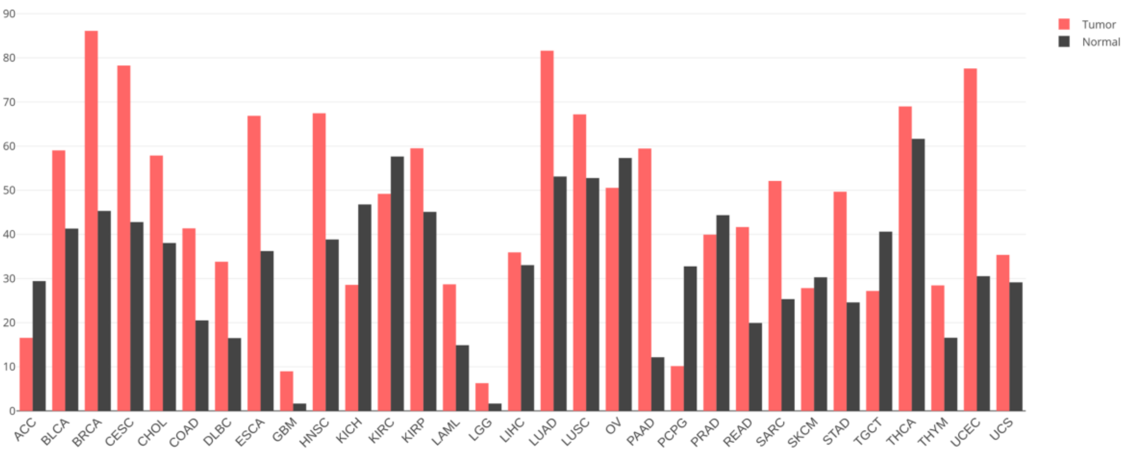

Figure S6. PanCancer analysis of B4GALT1 expression. The barchart represents the B4GALT1 expression in 31 cancer types and the relative normal (red = cancer; grey = normal). Abbreviations = ACC Adrenocortical carcinoma; BLCA Bladder Urothelial Carcinoma; BRCA Breast invasive carcinoma; CESC Cervical squamous cell carcinoma and endocervical adenocarcinoma; CHOL Cholangio carcinoma; COAD Colon adenocarcinoma; DLBC Lymphoid Neoplasm Diffuse Large B-cell Lymphoma; ESCA Esophageal carcinoma; GBM Glioblastoma multiforme; HNSC Head and Neck squamous cell carcinoma; KICH Kidney Chromophobe; KIRC Kidney renal clear cell carcinoma; KIRP Kidney renal papillary cell carcinoma; LAML Acute Myeloid Leukemia; LGG Brain Lower Grade Glioma; LIHC Liver hepatocellular carcinoma; LUAD Lung adenocarcinoma; LUSC Lung squamous cell carcinoma; MESO Mesothelioma; OV Ovarian serous cystadenocarcinoma; PAAD Pancreatic adenocarcinoma; PCPG Pheochromocytoma and Paraganglioma; PRAD Prostate adenocarcinoma; READ Rectum adenocarcinoma; SARC Sarcoma; SKCM Skin Cutaneous Melanoma; STAD Stomach adenocarcinoma; TGCT Testicular Germ Cell Tumors; THCA Thyroid carcinoma; THYM Thymoma; UCEC Uterine Corpus Endometrial Carcinoma; UCS Uterine Carcinosarcoma; UVM Uveal Melanoma.

MSigDB:HALLMARK (N=1241, FDR<0.001 & B> 0.3)

**EPITHELIAL MESENCHYMAL TRANSITION**	FN1 SERPINE1 IGFBP3 TGFBI LAMC1 SPP1 TFPI2 SPOCK1 TIMP1 QSOX1 EDIL3 ECM1 HTRA1 VEGFC PLAUR GJA1 IGFBP4 LRP1 NT5E MMP1 TNFAIP3
**TNFA SIGNALING VIA NFKB**	TNFAIP3 PTGS2 CCL20 NFKB2 BIRC3 PLAUR SOD2 NR4A1 SERPINE1 TNFAIP6 KYNU G0S2 BTG1 KLF10 LAMB3 B4GALT1 PDE4B DUSP4
**HYPOXIA**	P4HA1 P4HA2 CCNG2 SERPINE1 DDIT4 IGFBP3 CITED2 ISG20 WSB1 STC1 BTG1 PLAUR PPP1R3C TNFAIP3 CP PAM TGFBI AKAP12
**COMPLEMENT**	SERPINE1 LGMN TIMP1 CTSB PLAUR DUSP6 FN1 LRP1 CTSD PLAT CALM1 KYNU CD46 CD55 CP TNFAIP3 TFPI2
**KRAS SIGNALING UP**	DUSP6 G0S2 BIRC3 TFPI TNFAIP3 IGFBP3 SPP1 TMEM100 PLAUR AKAP12 PLAT CCL20 PTGS2 ANKH VWA5A
**GLYCOLYSIS**	P4HA1 IGFBP3 CITED2 QSOX1 SPAG4 P4HA2 B4GALT1 DDIT4 GLCE STC1 ISG20 PAM NT5E TGFBI CTH
**COAGULATION**	SERPINE1 MMP1 FURIN LGMN CTSB TIMP1 PLAT FN1 TFPI2 HTRA1 LRP1 DUSP6
**CHOLESTEROL HOMEOSTASIS**	FDPS IDI1 ACSS2 TRIB3 LGMN ANTXR2 STARD4 PLAUR
**P53 PATHWAY**	ZMAT3 DDIT4 BTG1 TXNIP ITGB4 TRIB3 VWA5A CTSD GM2A RPS12 APP
**APOPTOSIS**	BIRC3 TIMP1 PLAT APP SOD2 CTH TXNIP ANKH ISG20

Table S1. Upregulated genes in 3D vs. 2D associated with most significant MSigDB: HALLMARKs.
